# Leading during change: the effects of leader behavior on sickness absence in a Norwegian health trust

**DOI:** 10.1186/1471-2458-12-799

**Published:** 2012-09-17

**Authors:** Vilde Hoff Bernstrøm, Lars Erik Kjekshus

**Affiliations:** 1 Department of Health Management and Health Economics, Institute of Health and Society, University of Oslo, Forskningsveien 3a, 0373, Oslo, Norway

**Keywords:** Sickness absence, Leadership, Social support, Loyalty, Problem confrontation, Negative leader behavior, Task monitoring, Organizational change, Restructuring, Health care

## Abstract

**Background:**

Organizational change often leads to negative employee outcomes such as increased absence. Because change is also often inevitable, it is important to know how these negative outcomes could be reduced. This study investigates how the line manager’s behavior relates to sickness absence in a Norwegian health trust during major restructuring.

**Methods:**

Leader behavior was measured by questionnaire, where employees assessed their line manager’s behavior (N = 1008; response rate 40%). Data on sickness absence were provided at department level (N = 35) and were measured at two times. Analyses were primarily conducted using linear regression; leader behavior was aggregated and weighted by department size.

**Results:**

The results show a relationship between several leader behaviors and sickness absence. The line managers’ display of loyalty to their superiors was related to higher sickness absence; whereas task monitoring was related to lower absence. Social support was related to higher sickness absence. However, the effect of social support was no longer significant when the line manager also displayed high levels of problem confrontation.

**Conclusions:**

The findings clearly support the line manager’s importance for employee sickness absence during organizational change. We conclude that more awareness concerning the manager’s role in change processes is needed.

## Background

When planning and executing change, many aspects must be considered, not only the possible benefits of a desired future state, but also how change will affect employees. Employees often experience change processes as stressful and threatening, and organizational change may lead to an increase in health problems [1] and sickness absence [2-4]. In the Norwegian healthcare sector, increasing demands for more services, higher quality, and better cost-effectiveness make change necessary. The question is therefore how to implement those changes with as little strain on employees as possible. To minimize strain on employees, we need more knowledge about factors that influence how individuals experience change.

The importance of leadership during organizational change is recognized in the literature. However, the emphasis is generally on the importance of leadership in ensuring the successful implementation of organizational change [5-8], and seldom on the importance of leadership for subordinates’ health and wellbeing. We expect that leaders also affect employees’ reactions to change and employees’ level of sickness absence. This study focuses on line managers as an important leaders during change, because they are the immediate superiors to implement and follow up any change actions. So far the line manager has been identified as a key source of change-related information to combat uncertainty among employees [9], and as an important source of social support during change [10]. Cummings, Hayduk, and Estabrooks concluded that the negative effects of hospital restructuring on employees were indeed reduced in resonant leadership environments, where the leader displays high levels of emotional intelligence [11].

Studies focusing on the effect of leader behavior in general have had similar findings [12-16].

This study investigates how the line manager’s behavior relates to employee sickness absence in a Norwegian health trust during major restructuring.

### How leaders may influence sickness absence

To comprehend how leader behavior might influence employee sickness absence, it is important to consider the complex nature of sickness absence. Sickness absence is here defined as all absence registered as absence due to ill health. Sickness absence is thus not a simple function of health, but an act influenced both by the employees’ motivation to attend work and by their ability to do so [17]. The leader may therefore influence sickness absence through different processes. On the one hand, the leader may increase the employees’ ability to attend by reducing the work demands that cause strain and impaired health for employees [18-21]. On the other hand, the leader may motivate employees to attend by providing them work resources, such as social support and feedback [18-21].

Bakker and colleagues looked at the total amount of absence and argued that the former process affected involuntary absence and the later voluntary absence [20]. We look only at sickness absence, and therefore assume that most measured absence includes a component of ill health [22,23]. Instead, we expect that the first process (demands) affects the employees’ ability to work and that the latter (resources) affects their choice to be absent given their present ability.

We also expect that the effect of leader behavior on employees will be particularly high during organizational change, making it an important time to investigate the relationship. Indeed, in the context of major restructuring, where the demands on employees are high, the employees’ available work resources are likely to become more important [24,25], and they might be more dependent on the leader to motivate and shield them from further work demands.

This study focuses on five common leader behaviors we expect to be particularly important for absence levels during change. We expect two of the behaviors to provide employees with increased resources (namely social support and task monitoring) and thereby to increase their motivation. The other three behaviors are ones we expect will influence the level of work demands experienced by employees, namely negative leadership, problem confrontation, and loyalty to superiors.

### The leader behaviors

*Social support*, or the leader’s display of care and encouragement, we expect to reduce sickness absence through increasing employee motivation. By fostering a positive social relationship and satisfying the employees’ need to belong, the leader gives employees an added reason to attend work [26,27]. Social support might also reduce sickness absence by buffering the effect of demands on health [28], for example, by giving emotional support or encouraging employees to see the situation in a more favorable light. Social support’s effect on reducing sickness absence is partially supported by the literature [13,29-31], though some studies have also found the opposite effect [e.g., [10,32,33].

*Task monitoring* measures to what extent the leader follows up on tasks to ensure they are done correctly. A leader who practices more task monitoring will have better opportunity to motivate employees by providing feedback on how the employees are executing their tasks. It is indeed a prerequisite that employees know how they are doing so that they feel the satisfaction of doing well and are motivated to do well in the future [34]. Proper feedback is also essential for employees to be able to improve and to achieve a desired feeling of competence [27]. Such feelings of pride and appreciation about a job well done might be important factors for intrinsic motivation [35]. Additionally, increased monitoring might also make unwarranted absence more visible to the leader and subsequently more uncomfortable for the employee.

*Negative leader behavior* concerns the leader’s display of bad behavior towards others, especially subordinates. He or she breaks the codes of what is considered good conduct towards others. Favoritism, pigeonholing, and blaming others for one’s own mistakes are examples of such bad behavior. Negative leader behavior is then distinct from simply the absence of positive behaviors. In comparison with previous literature, our definition of negative leader behavior is similar to what Higgs terms “inflicting damage on others” [5], and to some extent to the “callous leader” Kellerman describes as uncaring and unkind [36]. Negative leader behavior is likely to lead to a more psychologically demanding relationship between employees and their leader, increasing the strain experienced by employees. Particularly when employees are already experiencing high demands caused by the change process, the increased strain caused by a difficult relationship with their superior will negatively affect their health and thereby increase their sickness absence. Though research is scarce, some previous findings show that negative leader behavior is associated with negative employee outcomes such as reduced motivation, increased stress, and increased sickness absence [5,37].

*Problem confrontation* measures to what extent the leader addresses problems when they arise and with those concerned. Problems in the work environment, such as conflicts among employees, will likely represent psychological demands on them. It is thus important for the leader to be willing to confront such problems in an effort to manage or resolve them. This expectation is partially supported by two studies: one by Reuver and Woerkom [38], who showed that a non-confrontational style to conflict management was related to lower employee commitment, and that low employee commitment was related to higher absence; and one by Saksvik and colleagues [39], who showed that the line manager’s focus on constructive conflict was important for healthy change. Additionally, addressing problems might also include addressing problems concerning high absences, making it more uncomfortable to be absent without good reason.

*Loyalty to superiors* concerns the display of loyalty and respect by line managers towards their immediate superiors. We propose that whenever change is executed from the top down, the less loyal line manager will have more room to reduce the demands put on employees by being more selective about when and how to implement directives from top management. The leaders will thereby reduce sickness absence by reducing the strain put on employees. A study by Choi and colleagues [40] found that a line manager’s sole attention to the mandate from top management hindered integration among employees after a merger.

In addition to the individual behaviors, we also consider *interaction effects*. We propose that different forms of leader behaviors should be analyzed not only independently, but also in interaction with each other. As argued by Aguinis and Gottfredsom [41], interaction effects are important for theoretical development in organizational science. Most likely, the employees’ interpretation and experience of one behavior are highly influenced by their general perception of their line manager and by other behaviors displayed by that manager. We therefore argue that to better explain the effect of one leader behavior, we also must account for how that leaderbehavior interacts with other behaviors. Previous research has indicated that the effect of social support on sickness absence is particularly influenced by the relationship and context it occurs in [10,32,33].

### The health trust and the change process

To investigate the relationship between leader behavior and sickness absence during organizational change, we study a large Norwegian health trust undergoing major restructuring, including a move to new premises and the implementation of a new organizational structure. The motives for the change process were several. An outdated hospital building brought about the need for new facilities, which in turn promoted investments in new, more modern equipment and procedures. The hospital sought to become the most modern hospital in northern Europe regarding such things as technical equipment, using robots, using new digital solutions for medications, and speech recognition in medical reports. An increase in the catchment area facilitated the need for growth and improved efficacy. Simultaneously, the new hospital facilities were seen as an opportunity to make further changes to the hospital’s organizational structure in an attempt to improve patient treatment and the use of personnel.

The move to new premises occurred in October 2008, while the planning and implementation of the new structure started already in 2005. The new organizational structure was inspired by the Mayo Clinic in the United States, and represented a radical new way of organizing the clinics. An important feature of the new structure was the establishment of a separate nurse division in January 2008. The nurse division organized all in-bed patients; consequently, traditional dividing of surgical and medical beds ceased to exist. The departments in the medical and surgical divisions no longer had their fixed number of beds available, but rather had to share available capacity. This way of organizing the hospital beds was called “flexible beds,” and was the most disputed change in the new hospital. An important rationale for flexible beds was that the concept offered a more effective way of organizing the beds. By implementing flexible beds the hospital administration hoped to avoid the need that some departments had to place patients in the hospital corridors although vacant beds existed in other departments. A consequence of “flexible beds” was that it increased the nurses’ responsibilities and control while reducing the physicians’ influence on bed management.

Additionally, other changes to the hospital’s organizational structure were made, such as merging previous departments into centers, in an attempt to improve collaboration (e.g., a center for laboratory work and a center for picture diagnostics). An important characteristic of the change process was that downsizing was not an issue.

The change process was characterized by an attempt to have employees actively participate in the change. Organizational development (OD) projects were created to contribute to the change process. The OD projects were largely made up of representatives from the different employee groups and were organized by a management team with three employee representatives. However, not everyone prioritized attending OD project meetings, as attending took time away for daily practices. Additionally, getting the line managers to promote the changes was challenging. Indeed, some line managers reportedly encouraged resistance to change.

## Method

### Participants

#### The health trust

The organization is a large Norwegian health trust with more than 4 000 employees spread over several locations. When the questionnaire was executed, February 2008, the health trust was in the starting phase of organizational change, as described above. Though this study looks at some specific changes occurring in 2008, it is important to add that the health trust had been in a state of change for quite some time prior to the study period.

#### The individual participants

The individual respondents to the questionnaire were employees from 35 departments at three divisions (surgery, medicine, and nursing) and two support centers (laboratory and picture diagnostics). The departments were selected because they were all influenced by the changes and were of an appropriate size to maintain anonymity. As previously mentioned, the two centers were created during the restructuring by merging smaller units. The division of nursing was created by moving tasks and responsibilities relating to hospital beds from the surgical and medical units into the new division. In this way, the new organizational structure particularly affected the three divisions. Among the respondents to the questionnaire, 86% were female, about 10% had managerial responsibilities, about 14% were doctors, and 45% were nurses or midwives. Participants ranged in age from 21 to 69, with an average age of 44. In total, 62% of respondents worked full time (37.5 hours a week or more) and 11% worked less than 70%. Table 1 presents more information about the individuals. All participation was voluntary.

**Table 1:** Descriptive statistic

	**Total**	**Div 1**	**Div 5**	**Div 2**	**Div 3**	**Div 4**
	**mean (SD) / %**	**mean (SD) / %**	**mean (SD) / %**	**mean (SD) / %**	**mean (SD) / %**	**mean (SD) / %**
Number of departments	17	10	7	6	6	6
Number of employees	2539	694	297	239	126	1183
Response rate	40%	37%	41%	51%	51%	38%
Female	86%	76%	76%	86%	83%	95%
Age	43.8 (11)	45.2 (10)	42.7 (11)	45.1 (10)	44.3 (11)	42.9 (11)
Employed full time	62%	67%	65%	74%	77%	53%
Employed 70–99%	27%	25%	20%	20%	17%	34%
Employed <70%	11%	8%	16%	6%	6%	13%
Nurse/midwife	45%	55%	22%	1%	2%	64%
Doctor	14%	22%	43%	17%	17%	0%
Assistant Nurse	9%	5%	5%	0%	9%	14%
Engineer	9%	0%	6%	61%	6%	0%
Radiographer	2%	0%	0%	0%	36%	0%
Managerial responsibilities	10%	12%	13%	13%	14%	7%
Sickness absence						
Absence time 1	6.4 (3.8)	6.9 (3.3)	4.2 (4.4)	4.5 (1.8)	7.2 (5.1)	9.1 (2.4)
Self-certified	0.8 (0.4)	0.8 (0.2)	0.4 (0.3)	0.9 (0.5)	1.0 (0.4)	1.1 (0.2)
Short medical certified	0.9 (0.7)	0.7 (0.5)	0.5 (0.5)	0.8 (0.6)	1.2 (1.3)	1.4 (0.3)
Long medical certified	4.7 (3.1)	5.4 (2.7)	3.3 (4.0)	2.8 (1.0)	5.0 (3.9)	6.6 (2.2)
Absence time 2	6.8 (4.0)	6.2 (3.1)	4.3 (3.9)	6.4 (3.3)	8.5 (5.6)	9.1 (3.2)
Self-certified	1.0 (0.5)	0.9 (0.5)	0.8 (0.3)	1.0 (0.5)	1.4 (0.5)	1.2 (0.4)
Short medical certified	0.9 (0.5)	0.8 (0.4)	0.6 (0.4)	0.8 (0.4)	0.9 (0.6)	1.5 (0.4)
Long medical certified	4.8 (3.5)	4.5 (2.4)	3.0 (3.5)	4.6 (3.2)	6.2 (5.6)	6.4 (2.5)

### Measurement

#### Organizational questionnaire

To measure leader behavior employees were asked to asses their line manager’s behavior. The questionnaire used was developed in collaboration between the University of Oslo and SINTEF research institute as part of a healthcare leadership evaluation. The questionnaire was first developed as part of a top healthcare management program in Norway [42]. The leadership program participants were asked about leadership issues and were asked to grade different issues through focus group interviews. A first version of the questionnaire was then developed with 115 statements [43]. It was then tested in a pilot study at the top healthcare management program, and was used to sort out leadership profiles. Based on a factor analysis, the statements were sorted into eight leadership competencies and the number of statements was reduced to 90 and then tested again in a large survey performed in 2005 at the largest health trust in Norway, having more than 8 000 employees [43].

Based on experiences from 2005, the questionnaire was further developed, and was then used at another university hospital in 2008. It consisted of 91 items, formulated as statements, in addition to background variables. Participants were asked to evaluate their line manager’s behavior by assessing, on a 5-point Likert scale, to what degree they agreed with the statements, from 1 (“to a small extent”) to 5 (“to a large extent”). The respondents were also given the option to answer “do not know” or “not relevant.” The present study uses the data collected during the leadership evaluation in 2008; however, it uses only those questions relating to the line manager’s behavior towards employees. For a full list of questions used in the present study see Table 2.

**Table 2:** Items used and standardized regression weights

**Variable**	**Items**	**Standardized regression weights**
	My leader:	
Social support	asks me how I am doing.	0.911
	supports me when I need encouraging.	0.904
	gives me positive feedback.	0.877
	asks me how my work is going.	0.904
Task monitoring	makes sure that I focus on the most important tasks.	0.841
	makes sure that I execute my tasks in the manner in which we have agreed.	0.868
	oversees that I execute my tasks.	0.867
Negative leader behavior	displays favoritism.	0.754
	interrupts me when I talk.	0.664
	asks me to execute tasks that are meaningless.	0.559
	gets me in a bad mood.	0.803
	blames others.	0.829
	belittles my views.	0.734
	obsesses with meaningless details.	0.690
	pigeonhole people.	0.870
	talks behind peoples back.	0.795
Problem confrontation	addresses difficulties when necessary.	0.924
	addresses problems with those concerned.	0.868
	addresses difficulties when they arise.	0.907
Loyalty to superiors	loyally executes instructions from superiors.	0.688
	talks about superiors with respect.	0.861
	works well with superiors.	0.966

We created a scale for each leader behavior by taking the average of the items included for that specific behavior type (see Table 2 for a list of items). This was done for each individual. Aggregated department scales were then created by averaging the individual scales, giving five continuous variables ranging from 1 to 5. Finally, these variables were standardized for the linear analyses.

#### Sickness absence

The data on sickness absence were collected from the health trust’s own register. The unit of analysis was all registered sickness absence measured in days of absence, divided by man-days, giving a department average of days missed. The variables were standardized for the linear analyses. More precisely, the variable includes the number of weekdays (Monday–Friday) registered as missed due to own ill health, from day one and until the person returned or no longer qualified for sickness absence benefits. In cases of part-time sickness absence, when a full day was not missed, only the appropriate portion of the day was counted as missed (e.g., 25%, 50%, etc.).

Sickness absence was measured at two times, average sickness absence from 1–6 months after the survey, and average sickness absence 7–18 months after the survey. The times were chosen based on their relations to different parts of the change process. The questionnaire was administered shortly after the establishment of the nursing division as an independent unit, allowing for the use of flexible beds. Seven months after the questionnaire was administered, the health trust moved to its new premises. Due to concerns about the participants’ anonymity, the statistics on sickness absence were aggregated to department level by the health trust before they were given to the authors. We therefore aggregated employee responses to department level as well and analyzed differences in level of sickness absence between departments. Information on the distribution of sickness absence at the two time points is given in Table 1.

### Analyses: validation of the questionnaire

To extract appropriate latent variables from the data material, the data were randomly divided in two. An exploratory factor analysis was conducted on the first half and a confirmatory factor analysis was conducted on the second. The factors were also evaluated based on their theoretical meaningfulness and appropriateness for the study’s purpose.

#### Exploratory factor analysis

Exploratory factor analysis was conducted using Maximum Likelihood (ML) with oblique rotation. The initial analysis found 13 factors with an Eigenvalue above one (Kaiser’s criterion). Several criteria were then used to decide which items and factors to keep. Items were considered meaningful when their loading on the factor exceeded .40 e.g., [44] and had a difference between factors of > .20 e.g., [45]. A factor was kept if it had a Cronbach’s alpha of > 0.70 [46]. Theoretical understanding was used to evaluate whether the items and factors were meaningful. This approach resulted in ten factors with between two and nine items. Below we will focus on factors relating to leader behavior, presented in Table 3. The content of these factors is presented in the Table 2, together with the standardized regression weights of each item.

**Table 3:** Mean, SD, skewness, Cronbach's alpha and correlations for the scales

					**Cronbach’s**				
	**Scales**	**Mean**	**SD**	**Skewness**	**alpha**	**1**	**2**	**3**	**4**
Individual level (N = 1008)									
Leader evaluation									
1	Social support	3.38	1.24	−0.44	0.94				
2	Task monitoring	3.05	1.04	−0.13	0.89	0.67**			
3	Negative leader behavior	1.74	0.81	1.40	0.92	−0.58**	−0.38**		
4	Problem confrontation	3.62	1.11	−0.67	0.93	0.72**	0.68**	−0.59**	
5	Loyalty to superiors	4.14	0.77	−0.97	0.73	0.38**	0.35**	−0.35**	0.46**
Department level (N = 35)									
Leader evaluation									
1	Social support	3.39	.46	-.28					
2	Task monitoring	3.15	.44	.48		0.73**			
3	Negative leader behavior	1.71	.40	2.04		−0.51**	−0.31		
4	Problem confrontation	3.69	.48	-.70		0.75**	0.75**	−0.53**	
5	Loyalty to superiors	4.03	.34	−1.35		0.34*	0.33	−0.33	0.39*
Sickness absence									
Time 1		6.37	3.80	.23					
Time 2		6.77	3.96	.33					

Note. All variables are measured using a 5-point Likert scale. * p <0.05 ** p < 0.01.

#### Confirmatory factor analysis

On the second half of the data, the new model was tested with confirmatory factor analysis using structural equation modeling. Again, we assumed that the factors were correlated. Table 4 shows the results. The results suggest a good fit of the model, with a good CFI value (0.93) and an acceptable and stable RMSEA (0.06) [47].

**Table 4:** Goodness of fit indices for the model

**Index**		
CFI	0.926	
RMSEA	0.059	(LO 0.056 HI 0.063)
Chi-squared (d.f.)	1714	(620)
N = 505		

#### Internal homogeneity

The constructs’ internal consistency was measured using Cronbach’s alpha. All constructs had an alpha value above 0.70, indicating good internal consistency (see Table 3).

#### Skewness

We also tested for skewness in the data (see Table 3) [48]. At the department level, only negative leader behavior had a significant positive skew value, indicating that there were limited reports of negative leader behavior. Sickness absence, which often suffers from skewness at the individual level, is more normally distributed at the aggregated level used for analysis.

### Ethics

The study fully complied with known ethical standards, including the Helsinki Declaration. The participation in 2008 was based on informed consent. When the data were re-used in 2010, the authors developed a design to protect the employees’ interests, to ensure the employees’ anonymity, and to ensure that the study was in line with the informed consent from 2008. With this special emphasis on anonymity, the project was then approved by REK (Regional Ethical Committee) of Western Norway (date: 08.10.2010).

## Results

In all, 1008 employees answered the questionnaire, an estimated response rate of 40% (calculated by dividing the number of usable responses by the number of employees at the departments). The response rate was higher in the smaller divisions than in the larger. Table 1 shows information on the distribution of response rate and on demographic variables of the respondents. In all, the response rate was below average (48%; SD 22) for surveys [49]. The low rate might be due to the fact that questionnaires were administered by email and that most employees had limited access to email during the workday. Wave analysis was conducted controlling for response bias [50], comparing those who responded before a reminder was sent out (495) with those who responded after (413). No significant differences were found for any of the variables (p > 0.05). Additionally, we conducted archival analysis to investigate if respondents differed on demographic variables from nonrespondents [50]. There was no significant difference in gender distribution between respondents and nonrespondents. Chi-square tests comparing age cohorts showed that the youngest age group (below 29) had a significantly lower response rate (p < 0.05). However, among those employees aged 30 or older there were no significant difference in age between respondents and nonrespondents (p > 0.05).

Some data were missing for some respondents due to item nonapplicability or omission (17%). Little MCAR test [51] indicated that the data were not missing completely at random. To better control for possible error because of missing data, we used an expectation-maximization (EM) algorithm to compute estimates to replace missing data. The estimates were created before the scales were created and aggregated, and are included in all analyses. Important to keep in mind is that EM imputations, though a clear improvement over more traditional methods, will still tend to underestimate the standard errors [52].

### The effect of leader behavior on employee sickness absence

To investigate the relationship between leader behavior and employee sickness absence, all data were aggregated to department level and standardized. We used multiple linear regression to estimate the relationship between the average employee answer in a department and the level of sickness absence in that department at time 1 (1–6 months after the survey) and time 2 (7–18 months after the survey). The analyses were weighted by department size to avoid giving small departments disproportionately high influence. As part of the analyses, we also tested for possible interaction variables. We were unable to directly control for factors such as age, work tasks, and education, as these data were deemed as jeopardizing respondents’ anonymity. However, some differences between departments were indirectly controlled for by controlling for division affiliation. Preliminary analyses were conducted to investigate the possible gain from distinguishing between self-certified absence and medically certified absence. However, the explanatory variables seemed to explain approximately the same proportion of variance in both types of absence. To maximize data strength, all absence was therefore included in the analyses presented here. Tables 5 and 6 present the results.

**Table 5:** Predictors of sickness absence (time 1)

	**β**	**SE β**	**95% CI**		**VIF**
(Constant)	0.43				
Social support	0.69**	0.23	0.21	1.17	3.26
Problem confrontation	−0.19	0.28	−0.77	0.39	3.59
Social support * problem confrontation	−0.26	0.13	−0.53	0.01	1.15
Loyalty to superiors	0.36*	0.17	0.01	0.71	1.39
Negative leader behavior	0.37	0.20	−0.04	0.79	1.65
Task monitoring	−0.69**	0.23	−1.17	−0.20	3.50
division b	−0.35	0.33	−1.03	0.33	1.42
division c	−0.67	0.34	−1.37	0.03	1.31
division d	−0.50	0.42	−1.37	0.38	1.23
division e	0.16	0.24	−0.34	0.66	1.84

Note. The values are calculated based on average department scores.

The analysis is weighted based on department size.

CI = confidence interval, SE = standard error.

N = 35 R^2^ = 0.672.

* p < 0.05 **p < 0.01.

**Table 6:** Predictors of sickness absence (time 2)

	**β**	**SE β**	**95% CI**		**VIF**
(Constant)	0.21				
Social support	0.31	0.22	−0.15	0.78	3.33
Problem confrontation	−0.49	0.28	−1.06	0.08	3.68
Social support * problem confrontation	−0.34*	0.13	−0.62	−0.07	1.11
Loyalty to superiors	0.42*	0.17	0.08	0.76	1.44
Negative leader behavior	0.13	0.19	−0.27	0.52	1.60
Task monitoring	−0.32	0.22	−0.76	0.13	3.34
division b	−0.26	0.34	−0.95	0.43	1.41
division c	0.03	0.32	−0.63	0.68	1.30
division d	0.02	0.39	−0.78	0.83	1.23
division e	0.34	0.23	−0.13	0.81	1.77

Note. The values are calculated based on average department scores.

The analysis is weighted based on department size.

CI = confidence interval, SE = standard error.

N = 35 R^2^ = 0.637.

* p < 0.05 **p < 0.01.

At time 1, three leader behaviors were significantly related to sickness absence, as Table 5 shows. Task monitoring (β = −0.69 p < 0.01) was related to lower levels of absence, whereas loyalty to superiors (β = 0.36 p < 0.05) was related to higher absence. Social support was also related to higher absence (β = 0.69 p < 0.01). We identified an interaction between social support and problem confrontation, which was correlated to lower absence. However, the interaction did not remain significant when controlling for division affiliation (β = −0.26 p = 0.058).

It might, however, be argued that with a limited sample size (N = 35), we do not have enough statistical power to control for these variables. And because, as stated by Aguinis and Gottfredson [41], there is a high chance of making type II errors when looking for interaction effects, we will keep the interaction variable in the model. The interaction variable’s effect was estimated as ΔR^2^ = 0.05 (ΔR^2^ = 0.08 when we did not control for division).

At time 2, three leader behaviors were significantly related to sickness absence, as Table 6 shows. Loyalty to superiors (β = 0.42 p < 0.05) had a positive relationship to absence. The interaction variable between social support and problem confrontation (β = −0.34 p < 0.05) remained significant, even when controlling for division affiliation. The interaction variable’s effect was estimated to be ΔR^2^ = 0.10 (ΔR^2^ = 0.11 when division affiliation were not controlled for). These results show a clear improvement in explained variance after the interaction variable is included in the model.

In both regressions we see a high R^2^ (0.67 and 0.64), indicating that the independent variables explain a high proportion of the variation between departments. The residuals from both regressions showed a normal distribution.

Because several of the leader behaviors were highly correlated to each other, we also tested for multi-collinearity. No general agreement exists concerning what constitutes excessive multi-collinearity. It is common to regard VIF values greater than 10 as excessive [53], however some authors argue that values as low as 4, or even 2.5, can be troublesome [53,54]. In our regression, no variables have a VIF value above 4.

**Figure 1:**
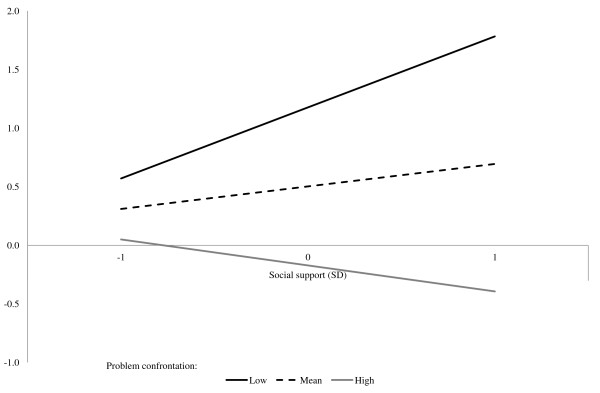
The relationship between social support and absence at different levels of problem confrontation (time 2).

**Figure 2:**
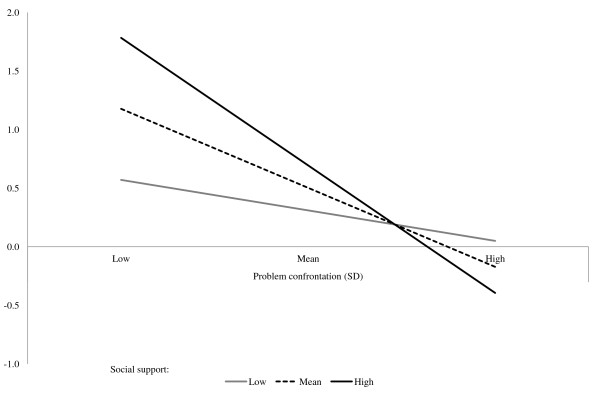
The relationship between problem confrontation and absence at different levels of social support (time 2).

It is important to investigate more closely the interaction between social support and problem confrontation to see what the estimated effect of one variable is at different levels of the other. We investigate the interaction by doing a simple slope analysis as described by Aiken and West [55]. Table 7 shows the results of the analysis, and Figures 1 and 2 illustrate the results for time 2. The analysis shows that social support is significantly related to higher absence at both times when problem confrontation is one standard deviation below average (β = 0.72 and β = 0.59 p < 0.05). Social support is not significantly related to absence at either time point when problem confrontation is one standard deviation above average. Problem confrontation is significantly related to lower absence at both times when social support is one standard deviation above average (β = −1.02 and β = −0.99 p < 0.01). Problem confrontation is not significantly related to absence at either time point when social support is one standard deviation above average. This analysis indicates that social support’s effect is no longer present when the leader also displays high levels of problem confrontation. Similarly, problem confrontation seems to be beneficial only if the leader also displays supportive behavior. 

**Table 7:** Simple slope analysis (times 1 and 2)

		** Absence time 1**		** Absence time 2**	
		**β**	**SE β**	**β**	**SE β**
Social support	Low problem confrontation	0.72*	0.32	0.59***	0.28
	High problem confrontation	0.02	0.31	−0.17	0.28
Problem confrontation	Low social support	−0.29	0.32	−0.20	0.28
	High social support	−1.02**	0.34	−0.99***	0.30

Note. Analysis is weighted based on the size of the departments.

SE = standard error.

N = 35.

* p < 0.05 ** p < 0.01 *.

## Discussion

### The effect of leader behavior on employee sickness absence

This study shows that much of the variance in sickness absence between departments during organizational change can be predicted by the line manager’s behavior prior to change. Four leader behaviors were related to employee sickness absence. However, contrary to our expectations, social support was related to higher, not lower, levels of absence.

Task monitoring was related to lower sickness absence at time 1. This finding supports the assumption that task monitoring provides managers with information they can use to give feedback to employees and to motivate them to attend work. The line managers’ display of loyalty to their superiors was related to higher sickness absence at both times. This result supports the assumption that the less loyal manager may reduce the demands experienced by employees during change by adapting the change process to employee needs. This finding is in line with the fact that several line managers indeed opposed the change process, and might have actively shielded their employees from the changes. Problem confrontation was related to lower sickness absence only in departments were the leader also displayed high social support. This finding partially supports the assumption of Saksvik and colleagues [39] that conflict resolution is an important part of healthy change. The line managers may reduce absence by confronting problems such as suspicious absence or conflicts that might strain employees. However, our results indicate that this strategy will be successful only if the leader is also seen as supportive. Negative leader behavior was not significantly related to sickness absence at any time.

Contrary to our assumptions, increased social support was related to increased sickness absence in departments that reported low and medium problem-confrontation behavior in their leader. Much empirical support exists for a relationship between low levels of social support and poor subjective health and high sickness absence e.g., [13,29-31]. However, a closer look at the literature suggests that the relationship is more complex. A Swedish study by Magnusson Hanson and colleagues [56] indicated that supervisor support was primarily important for men’s health, while women responded more to colleague support. Väänänen, Pahkin, Kalimo, and Buunk [32] found that supervisor support affected subjective health positively after a merger, but only among white-collar workers. Among blue-collar workers, strong social support from coworkers increased the effect of a decline in job position on subjective health. Terry and colleagues [10] found a direct positive effect of supervisory support on wellbeing, but an indirect negative effect of colleague support on wellbeing. Bacharach, Bamberger, and Biron [33] found when investigating the effects of alcohol consumption on absence that whereas high coworker support reduced the effects of high alcohol consumption on absence, high supervisor support seemed to increase the effect. They suggest that a supervisor who is perceived as supportive might also be perceived as more understanding and tolerant of absenteeism.

Setting our findings in the context of previous research, we argue that leader behavior is not experienced in a vacuum, but rather in the context of the relationship in which it occurs. Based on our findings and previous research, this interaction might be particularly true for social support. Considering the conclusions of Bacharach and colleagues [33] that social support might sometimes lead to increased sickness absence by causing employees to believe that absence is understood and accepted, we propose that this perceived tolerance can be maintained only when the supervisor does not address problems with absenteeism. In our study, social support was not related to increased absence in departments with leaders who practiced high problem confrontation. It is also possible that a line manager’s display of support and perceived tolerance of absence might be of extra importance in situations where added stress makes sickness absence a more accepted coping mechanism.

To sum up, this study’s results clearly highlight the importance of the line manager’s behavior for sickness absence during organizational change. The results also identify some specific leader behaviors as particularly important, and the necessity of interpreting the different leader behaviors in interaction with each other. To what extent these behaviors’ importance is restricted to settings of organizational restructuring should be further investigated in future studies.

### Sickness absence: A measure of ill health or motivation

In interpreting the findings, it is important to discuss what we measure when we study registered sickness absence. Registered sickness absence is all absence registered as absence due to ill health. As previously argued, sickness absence is not a simple function of health, but an act influenced both by the employees’ motivation to attend work and their ability to do so [17]. Research has indicated that the proportion of registered sickness absences that is unrelated to ill health is likely to be small [23]. However, while several diseases or injuries preclude the possibility of attending work, many also leave room for the individual to decide. Motivation therefore plays an important role in the individual’s decision whether to be absent or to attend work when ability to work has been reduced [22]. The relationship between leader behavior and sickness absence might therefore be mediated by both motivation and ill health.

Empirical research indicates that medically certified long-term absence is an accurate measure of ill health while self-certified absence to a greater extent is also influenced by other factors, such as job satisfaction and subjective experiences [57-59]. Our analysis shows that leader behavior explained variance of both medically certified absence and self-certified absence. These results therefore indicate that leader behavior might influence employee health as well as motivation. However, this finding should be further investigated in future research.

### Limitations

We must be careful in interpreting causal interferences between the variables, as we lack a control group and have only a limited number of control variables. By controlling for division affiliation, we attempt to indirectly control for departmental differences that could influence our results (e.g., demography, work task, and effect of change), but having a greater number of detailed control variables would have been beneficial. However, the fact that leader behavior and sickness absence are measured at different times is important, because such an approach largely protects us against making wrong assumptions about the direction of the causal relationship.

Due to anonymity concerns, it was necessary to perform the analyses at an aggregated level. We would have preferred to use alternative methods, such as multilevel analyses, as we lose information and the analyses become less efficient when we aggregate. Because we are not interested in individual differences, parts of the information lost are of less importance for the present study. Still, it is especially important to remember that absence of evidence (e.g., for the effect of negative leader behavior) is not evidence of absence [60].

When we aggregate the questionnaire responses, we also make assumptions about the respondents’ representativeness. Our dependent variable is the total absence level at each department, yet our independent variable (leader behavior) is based on the responses of 40% of the employees at the given departments. In the analyses, we therefore assume that the respondents adequately represent their entire departments. If the nonrespondents had answered significantly differently than the respondents did, there would have been a nonresponse bias [50]. In the case of nonresponse bias, it can be misleading to generalize the results to the population. A high response rate is generally considered important because it is expected to reduce the probability of nonresponse bias [61,62]. The response rate of the present study was 40%, which is below the average of 48% in published articles [49]. Attempts have been made to formulate general rules about acceptable levels of response rates (e.g., analyses done by Kramer, Schmalenberg, Brewer, Verran, and Keller-Unger showed that a response rate of 40% or more was adequate to obtain representative data [63]). However, such rules may be misleading, because data with high response rates may suffer from more nonresponse bias than data with low response rates does [50,61]. Instead, it is important to also use techniques that more directly attempt to assess nonresponse bias [50]. In the present study, we used archival analysis and wave analysis to look for possible signs of nonresponse bias. Though there is an underrepresentation of employees in the youngest age group (younger than 29 years old), the results generally support the representativeness of the respondents. It is nevertheless important to keep in mind that errors could arise because the samples used for the independent and dependent variables were different.

Aggregating leader behavior to department level also limits our ability to make conclusions at an individual level. Instead, conclusions are made at department level (e.g., the department where the leader displays high social support also suffers from higher absence. However, the individuals experiencing the highest social support might not be absent). This limitation may, however, also be viewed as a strength. For example, it is less likely that the results are caused by positive people who both are less absent and evaluate their leader more favorably.

Because the data had limited strength and lacked detail, we were unable to analyze more precisely leader behavior’s effect on different types of absence. We were unable to distinguish between short- and long-term absences because the data lacked strength. Dividing the dependent variable would have limited the variation in the variable, and it would have been harder to get significant findings with the present data’s limited sample size. Previous research has shown that many risk factors are the same for both long and short spells of absence, including several work characteristics [64]. However, that both short- and long-term absence absences are included means that a small proportion of employees with long spells of absence unproportionately affect the data and results. We were also unable to distinguish from the rest those diagnoses more likely to have been influenced by a change process and by leader behavior. Most medically certified absence is absence due to muscle and skeletal disorders and mental illness, covering 42% and 18% of all absence, respectively, in 2008 [65]. These diagnosis are both health problems typically associated with stress [66-68], which therefore is likely to be influenced by change processes and by leader behavior (as argued above). Furthermore, it is possible that a stressful work environment influences other types of sickness absence (e.g., pregnancy-related absence [69]), though the effect will likely be much smaller. Nevertheless, further analyses differentiating between diagnosis and / or length of absence might give important information about the nature of the relationship between leader behavior and sickness absence.

Finally, the study did not use a previously validated questionnaire to measure leader behavior; instead, we used data collected as part of a leader evaluation. Therefore, we paid extra attention to the survey’s psychometric properties, and tested the factor structure’s validity as part of the present study. The results have supported the assumption that the questionnaire is reliable and valid. In addition, because the questionnaire was developed especially for leader evaluation in a health care environment, it encompasses issues especially important for leaders in the healthcare sector and other important variables less frequently studied, such as loyalty to superiors and negative leader behavior. Therefore, the questionnaire provided a great opportunity to study potentially important aspects of the line manager’s behavior that have received limited attention in the literature so far.

Despite the present study’s limitations we believe that this paper is a valuable contribution to the research field. It can be difficult to obtain access to data in an organization experiencing major restructuring, and consequently, the topics discussed in this paper have received limited attention in the literature so far. It is therefore important to make the best use of the opportunity when data are available. By combining data from different sources, collected at multiple times, the present study gives important information about the importance of the line manger during organizational change.

### Generalizability and the uniqueness of the health sector

Norway’s health sector has many characteristics that differ from those of other sectors and of for-profit companies in ways that might influence the findings and their generalizability. These differences, however, also make it particularly important to study such public organizations.

First, Norway’s health sector is continually expanding, and at most large hospitals (including the one studied) the threat of downsizing is slim to nil. This security might reduce the strain on employees, but it might also remove some of the disciplinary function that the fear of losing one’s job represents. The importance of monitoring and the leaders’ willingness to address problems concerning suspicious absence might therefore both be heightened.

Second, hospitals have been described as organizations decoupled between top management and the medical staff [70]. The line managers are still an active part of the medical staff, and might therefore be expected to stay loyal to their fellow clinicians. The negative consequences of a line managers’ undivided loyalty to superiors might therefore be stronger in the health sector.

Third, registered sickness absence in health and social services is the highest in Norway, more than 2 percentage points above the average in all sectors [71]. Because the absence levels are consistently higher than are those in other sectors, other factors might influence absence levels in this sector. The high levels of absence might create more opportunities for the leader to have a visible influence than in sectors with minimal absence.

Finally, employee characteristics, particularly gender distribution, are an important aspect of the health sector. The vast majority of hospital employees are female. In the present study, 86% of participants were female. In Norway, females have both higher self-certified absence and higher medically certified absence, even after subtracting pregnancy-related absence [65,71]. A literature review of the relationship between sickness absence and gender showed that the psychosocial work environment might influence women’s sickness absence somewhat differently than men’s [72]. The review indicated, though not conclusively, that women might react differently to stressors, use different resources, and to a greater extent use absence as a coping mechanism. Active jobs, with high psychological demands and high control, have been associated with increased absence among women [30,59,73]. However, among men active jobs were not related to absence or were associated with decreased absence [30,59,73]. Similarly, an autocratic leader style was related to higher sickness absence in men, but not in women. And while women seemed to be adversely affected if their leaders practiced too much or too little team integration, men were not [12]. Perhaps, therefore, the results of the present study are not generalizable to a male-dominated population.

## Conclusions

We argue that this study’s findings clearly support the line manager’s importance for levels of sickness absence during organizational change. The findings also indicate that some leader behaviors are particularly important. This study should serve as a reminder that change is not executed by top management alone. Leaders in every organizational layer participate in shaping the result of the initiated changes, and thus line managers should be given room to adapt the changes to their departments.

Another important contribution of the study is to highlight the complexity of how leader behavior might influence employees. The findings show that employees’ reactions to one leader behavior might depend on other aspects of the leader-employee relationship. Leader behavior should be viewed as embedded in the relationship and in the situation in which it occurs, and should not be analyzed free of context.

## Competing interests

The authors declare that they have no competing interests.

## Authors’ contributions

LEK contributed to developing the questionnaire and to data acquisition. VHB contributed to data analysis and drafted the paper. Both authors contributed to substantially revising the paper. And both authors read and approved the final manuscript.

## Pre-publication history

The pre-publication history for this paper can be accessed here:

http://www.biomedcentral.com/1471-2458/12/799/prepub
